# The Fear of Smell: The Relationship Between Obsessive Traits and Self-odor Concern

**DOI:** 10.1192/j.eurpsy.2023.1969

**Published:** 2023-07-19

**Authors:** S. Tempia Valenta, C. Bronte, F. Panariello, F. Bonazzoli, D. De Ronchi, A. R. Atti

**Affiliations:** Department of Biomedical and Neuromotor Sciences, University of Bologna, Bologna, Italy

## Abstract

**Introduction:**

DSM-5’s framing of Obsessive-Compulsive and Related Disorders (OCRDs) paved the way for the increasingly structured definition of obsessive-compulsive spectrum disorders. The spectrum would include, among others, body dysmorphia, hair-pulling, skin-picking, obsessional jealousy, and olfactory reference syndrome (ORS). ORS – i.e., persistent concern about emitting a foul or offensive body odor – causes clinically significant distress or impairment in several areas of functioning.

**Objectives:**

This study aimed to investigate the relationship between obsessive traits and self-odor concern in a clinical sample that did not meet the diagnostic criteria for either OCRDs or ORS.

**Methods:**

In a sample of 220 adults referring to an outpatient Mental Health Service in Bologna, Northern Italy, we measured (1) self-odor concern through two specific items – sweat hatred (SH) and body odor hatred (BOH) – on the Body Uneasiness Test (BUT) and (2) obsessive traits through the total score of the Obsessive-Compulsive Inventory-Revised (OCI-R). Therefore, we performed correlation and regression analysis to examine the relationship between obsessive-compulsive traits and self-odor concern.

**Results:**

We found a positive correlation between OCI-R and SH scores (r = 0.330) and OCI-R and BOH scores (r = 0.188). Linear regression analysis demonstrated that OCI-R score significantly predicted SH score [F(1, 218) = 26.455, R^2^ = 0.109, p < 0.001] and BOH score [F(1, 218) = 8.017, R^2^ = 0.035, p = 0.005], highlighting that obsessive-compulsive traits predict both sweat and body odor hatred.

**Conclusions:**

These results demonstrate that obsessive traits and self-odor concern are strictly connected. This knowledge may allow us, even in the absence of an overt diagnosis of OCRDs or ORS, to better identify an at-risk population before it suffers impairment in functioning. Overall, further research is needed to help characterize obsessive-compulsive spectrum disorders before symptom exacerbation.

**Disclosure of Interest:**

None Declared

